# *TERT* Promoter Mutations Are Predictive of Aggressive Clinical Behavior in Patients with Spitzoid Melanocytic Neoplasms

**DOI:** 10.1038/srep11200

**Published:** 2015-06-10

**Authors:** Seungjae Lee, Raymond L. Barnhill, Reinhard Dummer, James Dalton, Jianrong Wu, Alberto Pappo, Armita Bahrami

**Affiliations:** 1 Department of Pathology, St. Jude Children’s Research Hospital, Memphis, Tennessee, 38105, USA; 2 Département de BioPathologie, Institut Curie, 26 rue d’Ulm, 75248, Paris cedex 05, France; 3 Department of Dermatology, University Hospital Zurich, Gloriastrasse 31, CH-8091 Zurich, Switzerland; 4 Department of Biostatistics, St. Jude Children’s Research Hospital, Memphis, Tennessee, 38105, USA; 5 Department of Oncology, St. Jude Children’s Research Hospital, Memphis, Tennessee 38105, USA

## Abstract

Spitzoid neoplasms constitute a morphologically distinct category of melanocytic tumors, encompassing Spitz nevus (benign), atypical Spitz tumor (intermediate malignant potential), and spitzoid melanoma (fully malignant). Currently, no reliable histopathological criteria or molecular marker is known to distinguish borderline from overtly malignant neoplasms. Because *TERT* promoter (*TERT*-p) mutations are common in inherently aggressive cutaneous conventional melanoma, we sought to evaluate their prognostic significance in spitzoid neoplasms. We analyzed tumors labeled as atypical Spitz tumor or spitzoid melanoma from 56 patients with available follow-up data for the association of *TERT*-p mutations, biallelic *CDKN2A* deletion, biallelic *PTEN* deletion, kinase fusions, *BRAF*/*NRAS* mutations, nodal status, and histopathological parameters with risk of hematogenous metastasis. Four patients died of disseminated disease and 52 patients were alive and disease free without extranodal metastasis (median follow-up, 32.5 months). We found *TERT*-p mutations in samples from the 4 patients who developed hematogenous metastasis but in none of tumors from patients who had favorable outcomes. Presence of *TERT*-p mutations was the most significant predictor of haematogenous dissemination (*P* < 0.0001) among variables analyzed. We conclude that *TERT-*p mutations identify a clinically high-risk subset of patients with spitzoid tumors. Application of *TERT-*p mutational assays for risk stratification in the clinic requires large-scale validation.

Spitzoid neoplasms are melanocytic tumors with distinct histologic characteristics that more commonly develop during the first 2 decades of life. Since their initial description by Sophie Spitz in1948^1^, the histologic diagnosis and appropriate management of spitzoid tumors have been controversial^2^,^3^,^4^,^5^,^6^,^7^,^8^.

Tumors with spitzoid morphology can present with a wide spectrum of biological properties, encompassing neoplasms that are entirely benign, called Spitz nevus, those with a low-grade or borderline malignant potential, termed atypical Spitz tumor (AST), and fully malignant neoplasms called spitzoid melanoma (SM). ASTs are tumors of intermediate malignancy that commonly spread to regional lymph nodes but do not progress to hematogenous metastasis^9^,^10^. On histological grounds, the distinction between an AST (tumors with metastatic capacity limited to regional lymph nodes) from SM (tumors with potential for extranodal metastasis) can be diagnostically challenging^3^. Sometimes lesions initially diagnosed as AST are reclassified as melanoma once distant metastasis develops. To date, no single histopathological criterion^7^ or molecular marker is known to predict with certainty the risk of subsequent aggressive disease with these tumors.

Telomerase activity is crucial for tumorigenesis and cancer progression^11^,^12^. The activity of telomerase, the enzyme responsible for maintaining telomeric DNA during replication, is regulated by the *telomerase reverse transcriptase* (*TERT*) gene^13^. Next-generation sequencing studies have identified somatic mutations in the core promoter region of *TERT* that by generating Ets/TCF transcription binding motifs increase the transcriptional activity of the gene^14^,^15^. *TERT* promoter (*TERT*-p) mutations have been found in 22%–71% of cutaneous melanoma in adult series^14^,^15^,^16^,^17^,^18^,^19^ and in the majority of conventional pediatric melanoma in a study from our group^20^, suggesting that they contribute to *TERT* regulation in melanoma. Interestingly, in our original series of pediatric melanoma, we found a hot-spot *TERT*-p mutation in the single patient with SM who died of disease but in no other patients with spitzoid tumors who had favorable outcomes^20^. We postulated that the molecular mechanism for maintaining telomere may be similar in SM and conventional melanoma. Herein, we investigated the presence of *TERT*-p mutations in 56 patients with histopathologically well-characterized atypical spitzoid neoplasms for whom follow-up information was available.

## Results

### Clinical Features

The clinical characteristics of patients are provided in Table S1. Tumors occurred in 33 female and 23 male patients aged 2–61 years (median, 9; mean, 14.6). They arose in skin of the lower extremity (*n* = 26), upper extremity (*n* = 9), face (*n* = 7), trunk (*n* = 6), ear (*n* = 5), and scalp (*n* = 3). Of the 42 patients who underwent sentinel lymph node evaluation, 21 (50%) had at least 1 positive lymph node, of which 9 had extensive nodal metastasis. Fifty-two patients were alive with no evidence of disease at last follow-up (mean, 32.5 months). Four patients developed hematogenous metastasis and died of widespread disease.

### Histologic Features

The histologic features of the 56 tumors are provided in Table S2. The Breslow tumor thickness ranged from 0.3 to 13.3 mm (median, 2.85 mm) in the 52 tumors with a favorable behavior and 1.3 to 8 mm (median, 5.25 mm) in the 4 tumors with an unfavorable behavior. The lesional diameter ranged from 1.5 to 17 mm (median, 6.5 mm) in tumors with a favorable behavior and 4 to 12 mm (median, 11 mm) for those with an unfavorable behavior. Ulceration was present in 10 of 52 (19%) tumors with a favorable and in 3 of 4 (75%) tumors with an unfavorable behavior. A high mitotic rate (>5/mm^2^) was seen in 5 of 52 (10%) tumors with a favorable behavior and in 3 of 4 (75%) tumors with an unfavorable behavior.

### *TERT* Promoter Mutations

**Figure 1:**
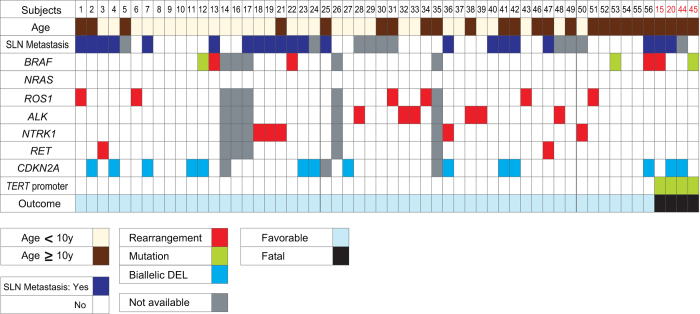
Association of kinase fusions, *BRAF* and *NRAS* mutations, biallelic *CDKN2A* deletion, and *TERT* promoter mutations with outcome in 56 patients with atypical spitzoid melanocytic neoplasm. Subject numbers in black font had a favorable clinical course and subject numbers in red font died of disseminated disease. Abbreviations: SLN, sentinel lymph node; DEL, deletion

Samples from 4 of 56 patients contained 1 of the known hot-spot single nucleotide variations (SNVs), including 3 SNV 228 G > A (C > T) (Chr5:1295228/hg19), at position −124 bp, and 1 tandem mutation 242/243 GG > AA (CC > TT) (Chr5:1295242–1295243), at positions −138/−139 bp from the ATG start site. The paired primary and metastatic tumor samples had similar variant mutations. All 4 patients with tumors harboring *TERT*-p mutations died from disseminated disease. In contrast, tumors in none of the 52 patients with a favorable clinical course carried these mutations (Fig. 1).

### Kinase Fusions

A panel of break-apart fluorescence *in situ* hybridization (FISH) for *ROS1*, *NTRK1*, *ALK*, *BRAF*, and *RET* was successfully performed in 51 tumors. Gene rearrangement was found in 23 of 51 (45%) tumors in mutually exclusive groups: *ALK* in 6 (12%), *ROS1* in 6 (12%), *NTRK1* in 5 (10%), *BRAF* in 4 (8%), and *RET* in 2 (4%). One of 4 tumors from patients with an unfavorable clinical course carried a *BRAF* fusion (Fig. 1).

### *BRAF* and *NRAS* Mutations

Of the 56 tumors, 3 (5%) carried a *BRAF* mutation (2 V600 E in patients with a favorable and 1 V600 K in a patient with an unfavorable clinical course). None of the tumors had an *NRAS* mutation (Fig. 1).

### *CDKN2A* (p16)

FISH identified biallelic *CDKN2A* deletion in 12 of 49 (24%) successfully tested samples from patients with a favorable clinical course and in 2 of 4 (50%) samples from patients with an unfavorable clinical course (Fig. 1). In some patients, the status of p16 was different between their primary and metastatic samples (see Discussion). As expected, biallelic *CDKN2A* deletion was predictive of loss of p16 by immunohistochemistry (Table S3). In 2 samples, immunohistochemical analysis showed loss of p16 expression without evidence of biallelic gene deletion by FISH (Table S3), suggesting that *CDKN2A* might be inactivated by mechanisms other than large deletion.

### *PTEN*

Biallelic deletion of *PTEN* was found in 4 of 40 (10%) samples from patients with a favorable clinical course and in none of the 3 successfully tested samples from patients with an unfavorable clinical course (Table 1).

### Association Analysis

The presence of *TERT*-p mutations was significantly associated with the risk of extranodal metastatic disease or death (*P* < 0.0001) (Table 1). In addition, age ≥10 years, mitotic rate  >5/mm^2^, and ulceration were each associated with the risk of extranodal metastasis or death (*P* <0.05). In contrast, no statistically significant association was found between the presence of biallelic loss of *CDKN2A* and extranodal metastasis or death (*P* = 0.56) (Table 1). Gender, nodal metastasis, primary tumor thickness, and tumor infiltrating lymphocytes were also not associated with extranodal metastasis. Lesional diameter was marginally associated with extranodal metastasis (*P* = 0.054). The presence of *TERT*-p mutations was correlated with age ≥10 years at diagnosis (*P* = 0.034), as well as mitotic rate  >5/mm^2^ and ulceration. A multiple-regression analysis to adjust the age effect was not feasible due to the small number of events (metastasis/death) in the cohort.

## Discussion

ASTs account for the majority of so-called melanomas encountered in children. Patients with atypical spitzoid neoplasms have frequent sentinel lymph node involvement, but their outcomes are much better than for patients with similar-staged conventional melanoma^21^,^22^. Lallas *et al.* conducted a systemic review of the literature and found that having positive sentinel lymph nodes did not predict a worse outcome in patients with AST^9^. In our study, 21 of 42 (50%) patients who underwent sentinel nodal sampling had positive nodes and 2 (5%) developed extranodal disease. Consistent with previous studies^9^,^22^,^23^,^24^,^25^,^26^, we find no correlation between regional nodal metastasis and subsequent development of aggressive disease in our patients (*P* = 0.61).

The difficulty in predicting the risk of hematogenous metastasis in patients with atypical spitzoid lesions has prompted extensive investigations. Sptaz *et al.* proposed a histopathologic grading scheme for risk stratification of pediatric AST^27^. We found a similar set of variables (age ≥10 years, ulceration, and mitotic activity  >5/mm^2^) associated with a later development of extranodal metastasis, although no single factor by itself was predictive of outcomes. Similar to the study by Heidenreich *et al.*^19^, the presence of *TERT*-p mutations in our cohort correlated with histopathologic parameters of poor prognosis, such as mitotic activity and ulceration. It is known that spitzoid tumors developing in the first decade of life (prepubertal ages), irrespective of having histologic attributes of malignancy, do not progress to distant metastasis, except in the rarest instances^21^,^28^. The absence of *TERT*-p mutations in tumors from younger patients (<10 years of age) in our study is consistent with this observation, although the underlying biological mechanism for the phenomenon remains to be elucidated.

Activated kinase signaling pathways via chromosomal translocations are responsible for tumorigenesis in spitzoid melanocytic tumors^29^,^30^. Wiesner *et al.* found kinase fusions of *NTRK1*, *ROS1*, *ALK*, *BRAF*, or *RET* in 55% of Spitz nevi, 56% of ASTs, and 39% of SMs^30^. Similarly, kinase fusions were found across the entire biological spectrum of spitzoid neoplasms in our cohort, indicating that they cannot be applied as a means to predict the biological behavior of spitzoid lesions.

The discovery of recurrent DNA copy number gains and losses in melanoma, and the absence of such changes in nevi, prompted the development of diagnostic assays to assist proper classification of histologically challenging melanocytic lesions^31^,^32^. The multiprobe melanoma FISH assay targets the common regions of alterations in melanoma: 6p25 (targeting *RREB1*), 6q23 (*MYB*), 11q13 (*CCND1*), and 9p21 (*CDKN2A*)^33^,^34^,^35^,^36^; whereas comparative genomic hybridization (CGH) studies provide a genome wide view of copy number changes^37^,^38^. Although both assays are valuable tools to discriminate nevi from borderline or malignant lesions, their ability in risk stratification for spitzoid tumors is uncertain. In our series, samples from 23 patients showed multiple copy number changes by CGH studies or an abnormal result on multicolor melanoma FISH assay, which supported an SM diagnosis, but only in 1 patient a clinically malignant phenotype became apparent (Table S3).

Biallelic 9p21 deletion is proposed as a marker for spitzoid tumors that are at high risk for aggressive behavior. In a study by Gerami *et al.*, of 37 pediatric patients with AST, 9 developed extensive locoregional disease and 2 developed distant metastasis. Tumors in 2 patients with distant metastasis and 7 of 9 patients with advanced locoregional disease harbored homozygous loss of 9p21, suggesting that this is a marker for disease progression^39^. We too observed the acquisition of biallelic deletion of *CDKN2A* (a reflection of tumor progression) in the nodal metastasis of at least 2 patients in whom the primary tumors retained both copies of the gene (Table S3), but neither patient developed extranodal metastasis in follow-up. As a whole in our series, the presence of biallelic *CDKN2A* deletion did not have a statistically significant association with the future risk of extranodal metastasis. Of the 4 patients who developed disseminated disease, 2 did not have biallellic *CDKN2A* loss in their primary or metastatic tumor and 2 had biallelic deletion in a subset of melanocytes that were not present at distant metastatic sites. Moreover, biallelic *CDKN2A* deletion was relatively common in the entire cohort, including in 23% of patients with a favorable clinical course. Overall, the findings from our study do not support that bialleclic 9p21 deletion is a reliable marker predictive of extranodal metastasis in spitzoid neoplasms.

Replicative immortality, a required element for overt malignant transformation in neoplastic cells, is commonly achieved by telomerase activation^11^,^12^,^13^,^40^. Several mechanisms are implicated in the control and reactivation of *TERT* in cancer cells, such as *TERT* gene copy gain or epigenetic modulation through *TERT*-p methylation^41^,^42^,^43^. In addition, mutations in the core promoter region of *TERT* have been shown to increase the transcriptional activity of telomerase and to be an independent marker of poor prognosis in various cancers, such as glioblastoma, thyroid carcinoma^44^,^45^,^46^,^47^,^48^, and cutaneous conventional melanoma^16^,^17^. The finding of *TERT*-p mutations in tumors from patients with fatal outcome in our series, and its absence in those with favorable behavior, strongly suggest that *TERT*-p mutations are a predictive marker of aggressive clinical behavior in patients with spitzoid lesions.

The negative predictive value of *TERT*-p mutations, on the other hand, requires further validation. *TERT*-p mutations may be acquired late during multistep melanoma development^49^. Conceivably, subclones of immortalized melanocytes with *TERT*-p mutations might be present in a lesion but be missed if below the detection level of standard sequencing assays. Moreover, the diverse mechanisms by which cancer cells maintain telomere length suggest that alternative mechanisms may be enacted alone or with *TERT*-p mutations to restore telomere length in melanocytes^41^,^42^. In addition, the follow-up time in our cohort was not extended enough to document the ultimate long-term outcomes of patients with wild-type *TERT*-p. One of our patients presented with disseminated disease 10 years after an initial diagnosis of AST (see Supplementary material). To prove the reliability and reproducibility of *TERT*-p mutations as a screening tool in the clinic, the assay needs to be evaluated on primary-site spitzoid neoplasms in large-scale studies with long term follow-up. We are currently collaborating with the Pediatric Melanoma Registry, an international multiinstitutional registry of pediatric melanoma based at the University of Pittsburgh, to work toward this aim.

The unpredictable clinical course of patients with spitzoid neoplasms has made the optimal management of these patients debatable, often leading to overtreatment of patients at minimal risk for disease progression. The use of *TERT*-p mutations as a marker of aggressive disease can help stratify a subgroup of patients with spitzoid tumors that likely behave in a malignant fashion and for whom more intensive therapies are warranted.

## Materials and methods

### Tissue Specimens

The study was approved by the institutional review board at St. Jude Children’s Research Hospital and the methods were carried in accordance with the approved guidelines. Written informed consent was not required under a HIPAA waiver IRB approval.

Tissue specimens from patients diagnosed with AST or SM were obtained from the surgical pathology archives. Inclusion criteria were as follows: (1) lesions showing some histologic features of Spitz nevus and meeting previously described criteria for AST and SM^4^,^20^,^21^,^50^; (2) availability of sufficient tissue for sequencing assays; and (3) availability of demographic and follow-up information. A comprehensive histologic evaluation by study investigators (RLB, RD, and AB) identified specimens from 56 patients with atypical spitzoid neoplasms (labeled as SM in 33 and AST in 23), including specimens from 5 previously reported patients^20^. The study material included formalin-fixed paraffin-embedded (FFPE) tissue specimens consisting of primary tumors (*n* = 49), paired primary and metastatic tumors (*n* = 5), and metastatic tumors (*n* = 2) from 56 patients.

### Histopathologic Parameters

The following histopathologic parameters were considered in the statistical analysis: (1) primary tumor Breslow thickness (T1 to T4); (2) horizontal lesional diameter (1–5 mm; 6–10 mm; >10 mm); (3) ulceration (present; absent); (4) mitotic rate (<1 per mm^2^; 1–5 per mm^2^; 6–10 mm^2^; >10 mm^2^); (5) tumor-infiltrating lymphocytes (absent; brisk; non-brisk); (6) regional lymph node metastasis (absent; small deposits; large deposits); (7) biallelic *CDKN2A* deletion (yes; no); (8) biallelic *PTEN* deletion (yes; no); and (8) *TERT*-p mutations (yes; no).

### Mutational Analysis of *BRAF*, *NRAS*, and *TERT* Promoter

FFPE tumor sections were manually microdissected guided by H&E slides to obtain at least 50% tumor purity in the material used for DNA extraction. Genomic DNA was extracted according to the manufacturer’s protocol using Maxwell® 16 FFPE Plus LEV DNA Purification Kit (Promega). Mutational hotspots for *BRAF* (exon 15), *NRAS* (exons 1 and 2), and a portion of *TERT*-p (HG19 coordinates, chr5: 1295151–1295347) were screened in genomic tumor DNA of the 56 tumors. PCRs were performed, using GoTaq® Long PCR Master Mix (Promega, Madison, WI) or AmpliTaq Gold® 360 Master Mix (Applied Biosystems, Foster City, CA) using amplification primers as previously described^20^. Direct sequencing of PCR products was performed using BigDye version 3.1 and a 3730XL DNA analyzer (Applied Biosystems, Foster City, CA). Results were screened using CLC Main Workbench sequence analysis software version 6.0.2 (CLC bio, Cambridge, MA).

### Fluorescence *in situ* Hybridization

BAC clones (BACPAC Resources, Oakland, CA) were used to develop copy number and break-apart probes for the following genes: *CDKN2A* (RP11-149I2) + 9q control (RP11-235C23), *PTEN* (RP11-2553L21) + 10p control (RP11-254A5 & RP11-322I2), *BRAF* (RP11-837G3 & RP11-948O19), *NTRK1* (CH17-67O18 & RP11-1038N13), *RET* (RP11-124O11 & RP11-718J13), *ROS1* (RP11-103F10 & RP11-1059G13), and *ALK* (CytoCell, Cat# LPS 019-A, Cambridge, UK). Dual-color FISH was applied on 4-μm FFPE sections as previously described^20^. FISH was successfully performed for a panel of kinase fusions (51 samples), copy number *CDKN2A* (58 primary/metastatic samples), and copy number *PTEN* (43 samples).

### Immunohistochemical Analysis

FFPE tumor sections were processed for immunohistochemical analysis for p16 (JC8; Santa Cruz) as previously described^20^.

### Statistical Analysis

Contingency tables were generated to study associations between outcome and risk factors. Pearson’s chi-square exact tests were used to test the associations.

## Additional Information

**How to cite this article**: Lee, S. *et al.* *TERT* Promoter Mutations Are Predictive of Aggressive Clinical Behavior in Patients with Spitzoid Melanocytic Neoplasms. *Sci. Rep.* **5**, 11200; doi: 10.1038/srep11200 (2015).

## Supplementary Material

Supplementary Material and Tables S1 and S2

Supplementary Table S3

## Figures and Tables

**Table 1:** Association of molecular markers and nodal status at diagnosis with outcome in 56 patients with atypical spitzoid melanocytic neoplasms (52 with favorable and 4 with unfavorable clinical outcome).

**Variables**	**Favorable Outcome (*n* = 52)**	**Unfavorable Outcome (*n* = 4)**
**Nodal metastasis at diagnosis** (*P* = 0.61)*		
Absent	20 (51%)	1 (33%)
Positive	19 (49%)	2 (67%)
NA	13	1
**Biallelic** ***CDKN2A*** **deletion** (*P* = 0.56)		
Yes	12 (24%)	2 (50%)
No	37 (76%)	2 (50%)
NA	3	0
**Biallelic** ***PTEN*** **deletion** (*P *= 1.00)		
Yes	4 (10%)	0 (0%)
No	36 (90%)	3 (100%)
NA	12	1
***TERT*****-p mutation** (*P* < 0.0001)		
Yes	0 (0%)	4 (100%)
No	52 (100%)	0 (0%)
**Oncogene** (*P* = 0.62)		
**Kinase fusion**		
*ALK*	6 (13%)	0 (0%)
*ROS1*	6 (13%)	0 (0%)
*NTRK1*	5 (11%)	0 (0%)
*RET*	2 (4%)	0 (0%)
*BRAF*	3 (6%)	1 (25%)
***BRAF*** **mutation**		
V600 E	2 (4%)	0 (0%)
V600 K	0 (0%)	1 (25%)
***NRAS*** **mutation**	0 (0%)	0 (0%)
**Not found**	23 (49%)	2 (50%)
**NA**	5	0

^*^P-values for association between each factor and risk of hematogenous metastasis/death
