# Supplementary material for: TERT Promoter Mutations Are Predictive of Aggressive Clinical Behavior in Patients with Spitzoid Melanocytic Neoplasms
Source: Sci Rep. 2015 Jun 10;5:11200. doi: 10.1038/srep11200 (PMC4462090; doi:10.1038/srep11200)
Supplement: Supplementary Material and Tables S1 and S2 [file srep11200-s1.doc]

***TERT* Promoter Mutations Are Predictive of Aggressive Clinical Behavior in Patients with Spitzoid Melanocytic Neoplasm**

Seungjae Lee^1^, Raymond L. Barnhill^2^, Reinhard Dummer^3^, James Dalton^1^, Jianrong Wu^4^, Alberto Pappo^5^, and Armita Bahrami^1^

Departments of ^1^Pathology, ^4^Biostatistics, and ^5^Oncology, St. Jude Children’s Research Hospital, Memphis, Tennessee 38105, USA

^2^Département de BioPathologie, Institut Curie, 26 rue d’Ulm, 75248, Paris cedex 05, France

^3^Department of Dermatology, University Hospital Zurich, Gloriastrasse 31, CH-8091 Zurich, Switzerland.

**SUPPLEMENTARY MATERIALS**

**Table S1. Demographic and Clinical Features for Patients with Atypical Spitzoid Neoplasm (52 with a Favorable and 4 with an Unfavorable Clinical Outcome)**

| **Clinical Features** | **Favorable (*n*=52)** | **Unfavorable (*n*=4)** |
| --- | --- | --- |
| **Age (years)** (*P* =0.03)*  <10  ≥10 | 31 (60%)  21 (40%) | 0 (0%)  4 (100%) |
| **Gender** (*P* =0.64)  Female  Male | 30 (60%)  22 (40%) | 3 (75%)  1 (25%) |
| **Location** (*P*=0.27)  Lower extremity  Upper extremity  Face  Ear  Scalp  Trunk | 25  9  6  5  2  5 | 1  0  1  0  1  1 |
| **Race** (*P*=0.70)  White  Black  Other  Not specified | 30 (81%)  2 (5%)  5 (14%)  15 | 4 (100%)  0 (0%)  0 (0%)  0 |
| **Lesional diameter** (*P*=0.054)  ≤5 mm  6-10 mm  >10 mm  NA | 22 (43%)  23 (45%)  6 (12%)  1 | 1 (33%)  0 (0%)  2 (67%)  1 |

* *P*-values for association between each factor and extranodal metastasis/death.

**Table S2. Histologic Features for the 56 Atypical Spitzoid Neoplasms**

| **Histologic Features** | **Favorable (*n*=52)** | **Unfavorable (*n*=4)** |
| --- | --- | --- |
| **Breslow thickness** (*P* =0.92)  T1 (<=1 mm)  T2 (1.01-2 mm)  T3 (2.01-4 mm)  T4 (>4 mm) | 8 (15%)  9 (17%)  18 (35%)  17 (33%) | 0 (0%)  1 (25%)  1 (25%)  2 (50%) |
| **Mitotic rate (per mm**^2^**)** (*P* =0.008)  1- ≤5  >5  NA | 46 (90%)  5 (10%)  1 | 1 (25%)  3 (75%)  0 |
| **Ulceration** (*P* =0.035)  Yes  No | 10 (19%)  42 (81%) | 3 (75%)  1 (25%) |
| **Tumor infiltrating lymphocytes** (*P* =0.72)  Brisk  Non-brisk  Absent  NA | 10  35  6  1 | 0  3  0  1 |
| **Nodal metastasis at diagnosis** (*P*=0.61)  Absent  Positive  Small deposits  Large deposits  Not performed | 20 (51%)  19 (49%)  10  9  13 | 1 (33%)  2 (67%)  1  1  1 |

* *P*-values for association between each factor and extranodal metastasis/death.

**The clinical course of 4 patients with an unfavorable outcome:**

***Patient no. 15:*** A 14-year-old white male was diagnosed with stage III spitzoid melanoma (SM) (T2N1M0; 2 microscopically positive sentinel lymph nodes). While on adjuvant interferon therapy, he developed lung, liver, and brain metastasis. He was treated with ipilimumab and temozolomide but died of disease 1.5 years after the initial diagnosis.

***Patient no. 20:*** Six months after an initial diagnosis of Spitz nevus on her thigh, an 11-year-old white female presented with a palpable inguinal node that was identified as a nodal metastatic melanoma. She received chemotherapy with cyclophosphamide, actinomycin, cisplatin, and interferon, followed by delayed nodal dissection, which demonstrated tumor deposits in 1 of 5 inguinal lymph nodes. One year later, she developed pulmonary metastatic disease that was treated with dimethyl triazeno imidazole carboxamide, tamoxifen, and interferon. She ultimately developed brain metastases and was treated with Taxol and radiation therapy but died 2 years after the initial diagnosis.

***Patient no. 44:*** A 61-year-old white female was first diagnosed with an atypical Spitz tumor (AST) on her nose (T3). Ten years after the diagnosis, on a routine visit she reported having vertigo for several months and a new subcutaneous nodule on her abdominal wall, the biopsy of which showed metastatic melanoma. Positron emission tomography–computed tomography and magnetic resonance imaging revealed pancreatic, rectosigmoidal, and brain metastasis. She was treated with 2 cycles of temozolomide and whole-brain radiation but died 7 months later.

***Patient no. 45:*** A 48-year-old white female had enlarged cervical nodes 1 year after the diagnosis of AST (T4aN0M0) in the occipital skin. Node dissection of the neck showed multiple positive lymph nodes and in-transit metastases. She developed progressive disease with new cervical and mediastinal nodes and pleural and pulmonary metastases while on vemurafinib (for 3 months), dacarbazine (1 cycle), cisplatin/vinblastine (3 months), and sorafenib and Taxol (3 months). She subsequently developed bone, skin, and brain metastasis and died 2 years after diagnosis.
